# Assessment of acquired apraxia of speech: pilot study with the CBO protocol

**DOI:** 10.1055/s-0046-1816038

**Published:** 2026-02-27

**Authors:** Beatriz Maurer Costa, Karin Zazo Ortiz

**Affiliations:** 1 Universidade Federal de São Paulo, Escola Paulista de Medicina, Departamento de Fonoaudiologia, São Paulo SP, Brazil.

**Keywords:** Apraxias, Articulation Disorders, Symptom Assessment, Diagnosis

## Abstract

**Background:**

Acquired apraxia of speech is often a comorbidity that accompanies aphasia. In some cases, it may be difficult to distinguish whether speech errors are phonological (resulting from aphasia) or phonetic (resulting from apraxia of speech).

**Objective:**

To verify the capacity of the Costa, Brescancini, and Ortiz (CBO) protocol to identify acquired apraxia of speech in persons with aphasia (PWA) among Brazilian Portuguese speakers.

**Methods:**

This is a cross-sectional and prospective study that included the participation of 7 PWA and suspected apraxia of speech (PWAG) poststroke and 25 neurotypical individuals who formed the control group (CG). All participants were followed the tasks of the CBO protocol, such as spontaneous conversation, description of a thematic card, word repetition, and diadochokinesias (DKK).

**Results:**

The protocol differentiated the groups in the spontaneous speech tasks (percentage of errors per word); word repetition list (time, punctuation, quantity, and type of manifestations); and DKK /ka/ and /pataka/. The results showed that the PWAG presented less fluent, slower speech, with more errors than the CG. Additionally, the protocol mapped the nature of the errors.

**Conclusion:**

The protocol enabled the identification of acquired apraxia of speech in PWA. It was also possible to analyze the tasks and linguistic variables that most interfered with the motor production of speech in Brazilian PWA.

## INTRODUCTION

Acquired apraxia of speech is one of the possible sequelae following a stroke. It is defined as a phonetic–motor disorder that impairs speech planning and programming. 1 It rarely occurs without aphasia. 2 Lesions in the frontal lobes, the left temporoparietal region, the insula, and the basal ganglia have been associated with cases of apraxia reported in the literature. 2 Stroke is the most common etiology of this disorder. 2

In their comprehensive review of the literature on apraxia of speech, McNeil et al. 3 identify specific manifestations, including intrusive schwa, a reduced speech rate that is often accompanied by the prolongation of vowels and/or consonants, as well as extended intervals between segments, which are characteristic of apraxia of speech. The authors also emphasize the significance of substitution errors, which may sometimes present as distortions, alongside variations in the stressed syllable of words, starters (false starts), attempts at the sound level, and general prosodic changes. 3

In 1997, Van Der Merwe proposed a speech model consisting of four stages: premotor or linguistic–symbolic planning, motor planning, motor programming, and motor execution. This model provided a more comprehensive description of motor speech production phases and clarified that motor planning deficits can lead to apraxia. 4 In 2021, the author revised this model and emphasized that apraxia of speech is a disorder of sensorimotor planning, highlighting the importance of spatial specifications (place and manner of articulation) and temporal specifications (related to interarticulatory synchronization) when analyzing the movements involved in sound production. 5

Incorrect specifications of spatial and temporal motor commands can hinder accurate sound production, leading to the distortion of one or more sounds in speech utterances, which may also lead to apparent substitutions. Slow recovery and increased planning load of longer, unfamiliar, or motorically complex utterances can contribute to slow speech, extended segmental and intersegmental durations, and speech produced syllable-by-syllable. The severity or frequency of speech errors may increase or decrease due to these contextual factors related to motor planning.

The manifestations observed in individuals with apraxia of speech and aphasia can be classified as phonetic-motor and phonological-linguistic, respectively. Some speech manifestations after the occurrence of a brain injury do not clearly show whether the failure is motor or linguistic. Therefore, they are conventionally called phonetic–phonological. For example, a substitution is a phonetically accurate production of a nontarget phoneme, with changes in at least two of the three characteristics: place, manner, and voicing features. This is typically considered a probable phonological-linguistic manifestation.

Meanwhile, distortion, that is, incorrect specifications of the spatial motor commands for the production of sounds, is considered a probably phonetic-motor manifestation. 6 On the other hand, addition and omission of phonemes, starters (false starts) at the syllable level and attempt at the syllable are considered phonetic–phonological manifestations. It is important to note that manifestations of substitution, omission, and addition of phonemes, classified as phonemic paraphasias in conduction aphasia, 2 cannot be attributed to either condition. Instead, they are manifestations that can have a phonological origin in aphasia and a phonetic origin in apraxia. Thus, accurate diagnosis depends on the correct characterization of the origin of each manifestation. Until 2019, sound distortion and prosody changes were the most used criteria in research on acquired apraxia of speech. 7

Despite significant advances in the identification of apraxia of speech characteristics, there is no standardized or universally accepted assessment tool currently available for this disorder. Moreover, because such evaluations rely on speech production, it is essential to consider the linguistic characteristics of each language. These factors highlight the need for the development and validation of speech-specific assessment instruments.

Given this difficulty, new protocols have been developed in recent decades to make the assessment process more effective in differentiating between language (aphasia) and speech (apraxia) disorders.

Among the protocols used to evaluate acquired apraxia of speech, the Apraxia Battery for Adults (ABA-2) 8 and the Apraxia of Speech Rating Scale (ASRS) 9 stand out, as they rely on the auditory perception of the speech–language therapist for speech assessment. In contrast, the Word Syllable Duration (WSD), 10 Pairwise Variability Index (PVI), 11 and Lexical Stress Ratio (LSR) 12 protocols use more objective acoustic measures, such as fundamental frequency and intensity.

In addition to these measures, diadochokinesias (DKK) are also used in the evaluation of acquired apraxia of speech, in which slower emission and greater difficulty in the production of /ka/ and /pataka/ are observed in this cohort. 13 14

With the publication of protocols for assessing acquired apraxia of speech, advancement in the understanding of motor planning has enabled the identification of variables that can hinder speech motor production. Regarding the assessment of apraxia, it is essential to control for variables such as word frequency, number of syllables, syllable frequency and structure, as well as the position of the stressed syllable in the word, as these interfere with motor planning. 15 Consequently, this affects the speech accuracy rate of individuals, 3 with errors becoming more evident in polysyllabic words. 16

In this sense, protocols for the assessment of apraxia that control such variables have become necessary, leading to the creation of new protocols to assess this disorder. Considering the phonetic–phonological differences among languages, a new protocol for Brazilian Portuguese has been proposed in 2024 by Costa, Brescancini, and Ortiz (CBO Protocol), 17 consisting of two lists of words, one for repetition and the other for reading aloud, while controlling for the identified variables. Additionally, the protocol includes tasks of spontaneous conversation, description of thematic cards, and DKK. 17 Although the CBO protocol controls for linguistic variables in the word list 17 that interfere in speech motor planning, such as frequency, word length, syllabic structure, and position of the stressed syllable, the protocol has not yet been used in individuals suspected of having apraxia of speech.

Considering that phonological alterations are expected in cases of aphasia, it is important to attempt to differentiate phonological (language-related) changes from phonetic (praxis-related) ones in these conditions. For this reason, we decided to study patients with poststroke aphasia. Thus, this study aimed to verify whether the CBO protocol for the assessment of acquired apraxia of speech is capable of accurately identifying this disorder, and whether variables that influence the motor production of speech—such as frequency, word length, and syllable structure—can contribute to a more precise identification of this condition in persons with aphasia (PWA) among Brazilian Portuguese speakers.

## METHODS

This is a cross-sectional pilot study developed at the Department of Speech-Language Pathology and Audiology of the Escola Paulista de Medicina (EPM), Universidade Federal de São Paulo (UNIFESP). The study was approved by the Research Ethics Committee of UNIFESP (0934P/2021), and participants signed a free and informed consent form, which was prepared according to the recommendations of the National Health Council in compliance with the resolution number 196 of October 10, 1996.

The PWA group (PWAG) included those with suspected apraxia of speech. In these criteria, the individuals should present at least one of the following manifestations: prolonged intervals between sounds; prolongations of sounds; starters (false starts) attempts at the sound level; and intrusion of schwa observed in spontaneous oral emissions ( Table 1 ). These patients suffered a single left-hemisphere stroke as the only neurological disorder, verified through medical records, and were assessed at the Acquired Neurologic Disturbances of Speech and Language Outpatient Clinic.

**Table 1:** The CBO protocol: 17 qualitative analysis of all speech tasks according to the classification adopted by Cera et al. 19 based on the criteria proposed by McNeil et al. 4

Phonetic/motor manifestations	Phonological/linguistic manifestations	Phonetic–phonological manifestations
Distortion: devoicing; delay in the beginning of voicing (e.g.: /'∫ʒorʒi/ for Jorge); or any other attempt with production deviating from the point, mode, or sonority than the correct production (e.g.: /'krasiku/ for “clássico”, /'tapo/ for “sapo”, /'su∫a/ for “xuxa”; /gre'naʒeη/ for “drenagem”; /Ri'ʒikulu/ for “ridículo”). Prolonged segments will be separated from the distortion. Prolonged interval between segments (phonemes, syllables, and words), such as /sa-‘pєka/ for “sapeca”. Vowel prolongation: emission time perceived as increased. Consonant prolongation: consonant emission time perceived as increased. Intrusion of *schwa* in consonant cluster: /’pedãra/ instead of “pedra”, /dere'naʒeη/ for “drenagem”. Intrusion of *schwa* between syllables: /sãn'pєka/ for “sapeca”. Sound rehearsal: search for the target sound with attempts emitted with separation from the final production, such as /'f-fãn/ for “fã”. Sound starter: repeated emission of phoneme without pause until the final production of the word, such as /'ffãn/ for “fã”.	Consonant substitution: exchange of one consonant phoneme for another, an error that involves a change in at least two of the three characteristics: point, mode, and sonority. Ex: /'Rifu/ for “riso”. Vowel substitution: exchange of one vowel phoneme for another. Self-correction: target production with spontaneous correction after the occurrence of a phonetic-phonological error (e.g.: /pe-prede-pedre'guλu/ for “pedregulho”). Perseveration: substitution in which there is a repeated occurrence of a phoneme that appeared previously in the word, ex: /kikan'deiru/ for “quitandeiro”. Anticipation: substitution in which there is the emission of a phoneme that would appear in the sequence of the word. Ex: /no'nita/ for “bonita”; /predre'guλu/ or /prede‘guλu/ for “pedregulho”. Transposition: substitution in which two phonemes are inverted in the word chain. Ex: /no’bita/ for “bonita”; /biRaŊ'seira/ for “ribanceira”; /t∫ikan'deiru/ for “quitandeiro”. Word rehearsal: search for the target word with attempts emitted with separation from the final production, such as /baŊ'kei/-/baŊ'keiru/ for “banqueiro” and /ga'ro/-/ga'rota/ for “garota”. Word starter: repeated emission of a word without pause until the final production, such as /kondo'miniukondo'miniu/ for “condomínio”.	Syllable starter: repeated emission of a syllable without pause until the final production of the word, such as /RiRi'dikulu/ for “ridículo”. Distorted substitution: deviated production of a non-target phoneme (e.g.: /'klas∫iku/ and /'klast∫iku/ for “clássico”; /'m:edu/ for dedo). Omission: absence of a phoneme or syllable from the word (e.g.: /'kasi/ for “classe”). Addition: addition of a phoneme or syllable to the word. Ex: /za'nõĩs/ for “anões” and /pedre'guλus/ for “pedregulho”. Syllable rehearsal: search for the target syllable with attempts emitted with separation from the final production, such as /baŊ-baŊ'keiru/ for “banqueiro” and /ga-ga'rota/ for “garota”. Syllable starter: repeated emission of a syllable without pause until the final production of the word, such as /RiRi'dikulu/ for “ridículo”.

Abbreviation: CBO, Costa, Brescancini, and Ortiz protocol.

The PWA underwent the Montreal-Toulouse Language-Assessment Brazilian Battery (MTL-BR) to identify language impairments supporting a diagnosis of aphasia. All of them were at chronic phase of aphasia (> 6 months when submitted to CBO protocol). 17

Finally, a control group (CG) was included, consisting of healthy individuals who responded to an invitation to participate in the research through social media.

The inclusion criteria for both groups were adults, 18-years-old, with more than 8 years of schooling. The exclusion criteria were previous history of speech and/or language disorders; prior stroke or aphasia; history of alcoholism or drug use; and auditory and/or visual disorders that could compromise performance during the application of the protocol.

The reading proficiency of the individuals was not a criterion for inclusion in the study. Although four of the seven individuals had the habit of reading before the lesion, only two were capable of doing it at the time of the evaluation. For this reason, the reading aloud task was not analyzed in this study.

The PWAG had 7 participants, and the GC had 25. All participants who met the inclusion criteria for participation in the research underwent the assessment tasks of the CBO protocol. 17 The tasks performed by all study participants and the analysis criteria are described in Table 2 . At the end of the analysis, the points obtained for each participant and each word were counted as described in Table 3 .

**Table 2:** The CBO protocol: 17 description of tasks and analysis criteria

**1. Spontaneous Conversation**	The participants answered 5 simple questions. They were analyzed according to the total number of errors per word emitted, and the percentage of errors was calculated.
**2. Description of a thematic card**	The participants were asked to tell a story about a bank robbery. 18 For this task, the number of errors per word emitted was analyzed, and the percentage of errors was calculated.
**3. Repetition of a list of words**	The participants had to repeat a phonologically balanced list consisting of 88 words of different extensions: monosyllabic, disyllabic, trisyllabic, and polysyllabic. They varied according to the frequency and position of each word's stressed syllable, as well as the number and structure of the syllables. The time considered was the total value obtained in seconds for completing the task. Two criteria were established to analyze this task: one quantitative ( Table 3 ) and one qualitative ( Table 1 ).
**4. DKK: of /pa/, /ta/, /ka/ and /pataka/**	The instruction was “Say the syllable /pa/ as fast as you can”. The same instruction was given for /ta/ and /ka/. Finally, the instruction was “Say pa-ta-ka as fast as you can”. The task was analyzed by marking the time and total syllable emissions in each DKK with Praat's Text Grids, 20 combining auditory perception and visual analysis of broadband spectrograms and intensity contours. 21 For each DKK, the total number of repetitions was divided by the total time, obtaining the value of emissions per second.

Abbreviations: CBO, Costa, Brescancini and Ortiz protocol; DKK, diadochokinesias.

**Table 3:** The CBO protocol: 17 quantitative analysis of the repetition list, criteria adapted from Dabul 10

Score	Description
0	response without hesitation, effort, or articulatory error;
1	a response that is recognizable as the target and has the correct number of syllables, but there is hesitation, effort, or articulatory error;
2	no response, an unrecognizable word, or a word with an incorrect number of syllables.

Abbreviations: CBO, Costa, Brescancini and Ortiz protocol.

Notes: Minimum score: 0, maximum: 176.

All participants were assessed individually by the same person, and all speech samples were recorded in an iPhone SE (Apple Inc.) 1st generation equipment for later analysis.

For the qualitative analysis of all speech tasks, incorrect emissions were analyzed according to the classification adopted by Cera et al. 18 in previous Brazilian Portuguese studies, based on the criteria proposed by McNeil et al., 3 presented in Table 1 . The occurrence of each type of error was computed.

### Statistical analysis

For the qualitative analysis, the errors from the first three tasks were transcribed, analyzed, and classified. Additionally, the results obtained in the word repetition task were analyzed according to the extension (monosyllabic, disyllabic, trisyllabic and polysyllabic), and syllabic complexity: consonant–vowel (CV), consonant–vowel–glide (CVG), consonant–consonant–vowel (CCV), consonant–vowel–consonant (CVC) and, consonant–vowel–consonant–consonant (CVCC). Furthermore, the word's frequency in the Portuguese language was analyzed based on the number of occurrences found in the Brazilian Corpus (PUC-SP), using the Linguateca tool. Then the descriptive analysis was carried out.

For the quantitative analysis of the groups, frequency tables and median were created based on the nature of the variables, and the Wilcoxon rank sum test was applied. A significance level of 5% was adopted for the test.

A logistic regression analysis was performed to assess the impact of age and years of schooling on the performance of the groups studied. Thus, the groups comprised the dependent variable, while age and schooling were the independent variables. All *p* -values below 0.05 were considered statistically significant, with a 95%CI.

The software used in the database analyses was R (R Foundation for Statistical Computing), 19 version 4.0.4.

## RESULTS

The sociodemographic data of the study groups are in Table 4 . There was a significant difference in the PWAG and CG groups, respectively, when comparing the age (45 versus 25-years-old; *p* = 0.004) and years of schooling (12 versus 16 years; *p* = 0.001) of the two groups. For this reason, logistic regression was applied, and it was found that age ( *p* = 0.114) and years of schooling ( *p* = 0.164) did not interfere with the analyses of interest in this study.

**Table 4:** Characterization of PWAG and CG according to sociodemographic data

Characteristics		PWAG	CG	*p* -value*
**Age: median (IQR)**		45.00 (42.00–49,50)	25.00 (22.00–32.00)	**0.004**
**Schooling: median (IQR)**		12.00 (11.00–13.00)	16.00 (14.00–16.00)	**0.001**
**Aphasia time (years): median (IQR)**		3.00 (2.00–5.00)	NA	
**Sex; n (%)**	Female	6 (86%)	19 (76%)	> 0.99
	Male	1 (14%)	6 (24%)	

Abbreviations: CG, control group; IQR, interquartile range; NA, not available; PWAG, persons-with-aphasia group.

Note: *Wilcoxon test.

Regarding the characterization of the PWAG, 4 individuals had mixed aphasia, 1 had mixed transcortical aphasia, 1 had Broca's aphasia, and 1 had anomic aphasia.

The quantitative analysis of each of the CBO protocol tasks can be seen in Table 5 . Both tasks of spontaneous conversation and the description of the thematic card were significantly different between the two groups ( *p* < 0.001). The total points (error score) and the total time to complete the repetition task were significant ( *p* < 0.001), when comparing the groups, as observed in Table 5 . In the Diadochokinesis task and only /ka/ ( *p* = 0.018) and /pataka/ ( *p* = 0.002) were statistically significant between the two groups.

**Table 5:** Quantitative comparison between the CG and PWAG groups in the CBO protocol tasks

Tasks	PWAG: median (IQR)	CG: median (IQR)	*p* -value
**Spontaneous conversation (% total number of words)**	8.33 (1.97–24.24)	0.0 (0.0–0.0)	**< 0.001**
**Description of a thematic card (% total number of words)**	5.88 (1.72–11.69)	0.0 (0.0–0.0)	**< 0.001**
**Repetition list**			
**Total points**	48 (41–82)	1 (0–2)	**< 0.001**
**Total time (s)**	301 (267–1,282)	183 (183–183)	**< 0.001**
**Diadochokinesia (number of syllables per second)**			
**Pa**	5.25 (4.61–5.78)	4.94 (4.51–5.34)	0.52
**Ta**	4.65 (3.93–5.59)	4.94 (4.65–5.77)	0.33
**Ka**	3.80 (3.76–4.25)	5.03 (4.32–5.44)	0.018*
**Pataka**	1.20 (0.59–1.37)	1.98 (1.75–2.16)	0.002*

Abbreviations: CBO, Costa, Brescancini and Ortiz protocol; CG, control group; IQR, interquartile range; PWAG, persons-with-aphasia group.

Note: *Statistically significant (Wilcoxon test).

**Figure 1:**
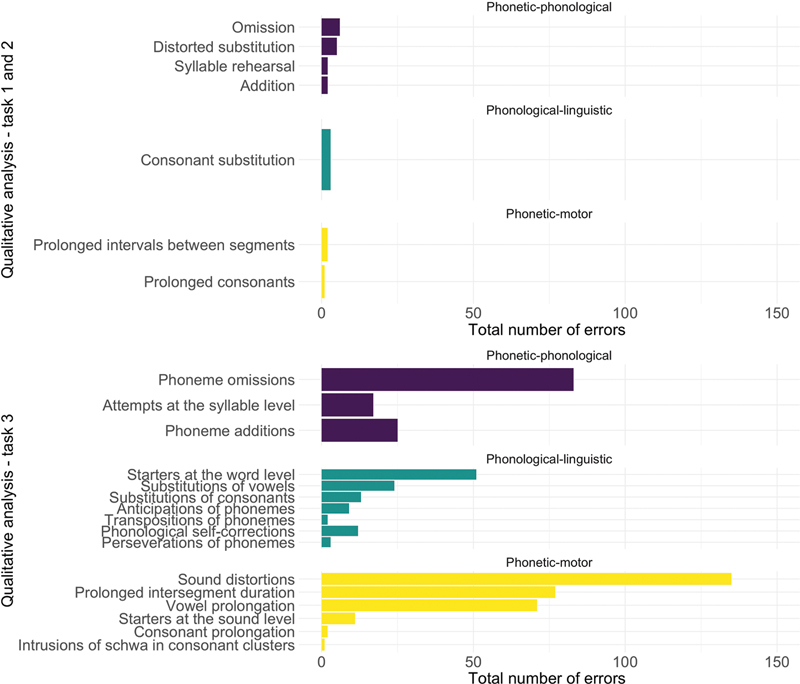
Number of manifestations committed by the persons-with-aphasia group (PWAG) in the tasks 1 to 3, grouped according to the possible origin of the error.

The qualitative analysis of the errors of the first 3 tasks is represented in Figure 1 . The graph was divided between the spontaneous speech tasks (task 1 and 2) and repetition (task 3). The control group only had syllabic intonation error, not predicted by the classification adopted in Table 1 .

**Figure 2:**
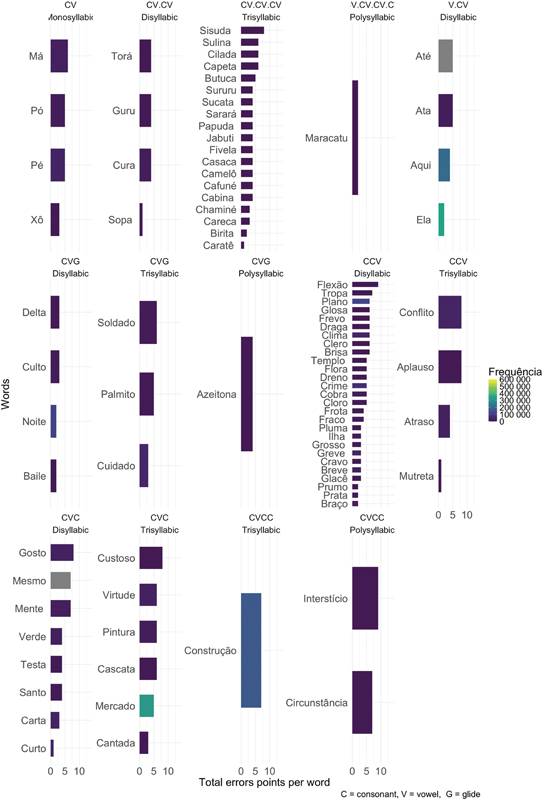
Performance of the PWAG group in the repetition list according to the total number of error points per word, considering the variables of interest.

Then, all the words in the list were analyzed for the total number of errors given by the score obtained in the PWAG group and were analyzed according to the variables of interest: extension, syllabic complexity, and frequency ( Figure 2 ). As the PWAG was composed of 7 participants, the maximum number of points per word was 14.

## DISCUSSION

The most relevant finding of this study was the significantly slower speech and more errors noted in the PWAG compared with the CG. It was possible to differentiate the two groups in the spontaneous speech tasks; repetition of the word list concerning time, punctuation (percentage of errors), quantity, and manifestation types; and the DKK /ka/ and /pataka/, since the control group registered almost no errors in these tasks. Therefore, the protocol allowed the identification of speech disorders and motor errors, highlighting the presence of apraxia of speech in PWA both in qualitative and quantitative analysis. Moreover, the test differentiated both groups and quantified errors in a widely reproducible way.

In spontaneous speech tasks, the PWAG had a median error percentage of 8.33% errors by the total number of words, while the GC had 0%. This difference was expected, but this measurement alone is not capable of clarifying the nature of the errors or distinguishing those originating from the linguistic system (aphasia) from the motor system (acquired apraxia of speech).

The speech tasks played a fundamental role in the analysis of errors and in the breakdown between phonetic and phonological errors.

Initially, the qualitative analysis of the manifestations that appeared in the spontaneous speech task demonstrated a predominance of errors that do not allow the determination of the origin of the speech disorder, motor (phonetic) or linguistic (phonological). The phonetic–phonological errors (omission and distorted substitution) corresponded to more than 50% of the manifestations, while phonetic–motor errors were the least prevalent (prolonged interval between segments and prolongation of consonants). This indicates the presence of manifestations suggestive of apraxia, but with less evidence.

However, in the repetition task ( Figure 1 ), motor errors became prominent, particularly in the form of distortion, prolonged intervals between segments, and prolonged vowels. This change in the types of errors is due to both the nature of the task and the characteristics of the repetition list. In connected speech, participants need to organize their responses based on their interpretation of the message, making it impossible to control for variables that may affect the motor production of speech. Additionally, the entire elaboration of the message is dependent on the linguistic system, with potential syntactic, semantic, and lexical alterations, typical of aphasia, as well as phonological alterations.

The repetition task had words with different complexities, making it more sensitive to identify alterations in motor planning than spontaneous speech tasks. Additionally, PWA may struggle to develop the content they need to express, leading to reduced spontaneous emission, which makes it difficult to conduct a comprehensive analysis of the manifestations and the variables that interfere with speech motor planning. This may lead less experienced evaluators to have an incomplete idea of the individual's condition, causing them to focus their investigation on aphasia based on the types of errors presented.

Thus, the repetition task becomes a valuable source for identifying difficulties and diagnosing apraxia of speech. This was observed in this study, where the errors identified in spontaneous speech were found to occur more frequently together with other manifestations in the repetition task. A comprehensive understanding of the nature of patients' difficulties enables more targeted and effective therapeutic guidance and, when examined longitudinally, provides a nuanced perspective on case progression.

The better performance of the CG in the repetition task corroborates research with Brazilian Portuguese speakers and others, 1 14 18 20 21 22 23 which report that, although neurotypical individuals can also respond differently to the demands of speech complexity. Also, they do not have difficulty in performing motor planning of words that differ in terms of length, syllable structure, and frequency. The repetition task facilitated the mapping of the nature of the errors, making it possible to identify the association of apraxia of speech with aphasia in PWAG due to the predominance of phonetic–motor manifestations, as observed in Figure 1 .

Figure 2 shows that, in the repetition task, the PWAG presented errors in all syllabic structures. However, the difficulties were more evident in words with complex syllabic structures, mainly CCV, CVC, trisyllables with simple CV structures, and low-frequency words in Brazilian Portuguese. This also corroborates the findings of other studies 14 15 22 in which greater difficulties are expected as word length and the complexity of syllabic structures increases, as well as in less frequent words. Therefore, the control of these variables is important in the investigation of changes in speech motor planning and can be useful for the development of speech therapies.

The protocol also demonstrated that the repetition tasks and the DKK /pataka/ better identified apraxia ( Table 5 ), especially due to the greater articulatory and coarticulation complexity of the phonemes, which makes the task more difficult.

Thus, the repetition task, with controlled stimuli as occurs in CBO, is capable of identifying the presence of apraxia through the nature of the errors. The PWAG presented slowed speech, which becomes more noticeable in repetition and DKK /pataka/. Worse performances in DKK 13 14 24 and prosodic changes in PWAG, such as prolonged interval between segments, prolonged consonant and vowel, rehearsal, and starter sounds, are in agreement with the literature. 1 10 13 14 This indicates that these tasks are useful to identify the presence of acquired apraxia of speech associated with aphasia.

Thus, the CBO protocol allowed the identification of apraxia of speech in spontaneous speech tasks (percentage of errors per word), especially in word repetition tasks (time, punctuation, quantity, and type of manifestations) and in the DKK /pataka/. The PWAG presented with slowed speech and more errors than the CG. The protocol identified speech manifestations according to the nature of the error, which helped in the differential diagnosis between apraxia of speech and phonemic paraphasias in PWA.

### Limitation to the study

This pilot study involved a small number of participants. For this reason, we suggest conducting further studies with larger sample sizes, specifically focusing on participants who can perform the reading aloud task. It is important to determine whether the performance in this protocol's reading task can differentiate between the groups (case and control) and if it is clinically equivalent to the findings obtained in the repetition task. Additionally, future studies conducted with PWA with and without suspected acquired apraxia of speech may provide clearer insights into which tasks and stimuli from the protocol are better in differentiating phonetic (praxis) from phonological (language) alterations.

Moreover, future studies could examine the application of the CBO protocol in cases of neurodegenerative diseases, such as Primary Progressive Apraxia, Primary Progressive Aphasia, Progressive Supranuclear Palsy, and Corticobasal Degeneration, as well as other syndromes associated with apraxia of speech. Such studies could also investigate the protocol's accuracy in identifying milder conditions, including Mild Cognitive Impairment. In conclusion, in this pilot study, the CBO protocol enabled the identification of apraxia of speech across all four tasks and demonstrated that the use of variables that influence motor planning aids in diagnosis. It also facilitated mapping of the number and nature of errors, a factor that can aid diagnostic accuracy, a key aspect for appropriate therapeutic intervention. This factor may also aid further studies.
